# Association between *H-RAS *T81C genetic polymorphism and gastrointestinal cancer risk: A population based case-control study in China

**DOI:** 10.1186/1471-2407-8-256

**Published:** 2008-09-10

**Authors:** Yongjing Zhang, Mingjuan Jin, Bing Liu, Xinyuan Ma, Kaiyan Yao, Qilong Li, Kun Chen

**Affiliations:** 1 Department of Epidemiology & Health Statistics, Zhejiang University School of Medicine, Hangzhou 310058, Zhejiang, PR China; 2 Institute of Cancer Research and Prevention of Jiashan County, Jiashan 314000, Zhejiang, PR China

## Abstract

**Background:**

Gastrointestinal cancer, such as gastric, colon and rectal cancer, is a major medical and economic burden worldwide. However, the exact mechanism of gastrointestinal cancer development still remains unclear. *RAS *genes have been elucidated as major participants in the development and progression of a series of human tumours and the single nucleotide polymorphism at *H-RAS *cDNA position 81 was demonstrated to contribute to the risks of bladder, oral and thyroid carcinoma. Therefore, we hypothesized that this polymorphisms in *H-RAS *could influence susceptibility to gastrointestinal cancer as well, and we conducted this study to test the hypothesis in Chinese population.

**Methods:**

A population based case-control study, including 296 cases with gastrointestinal cancer and 448 healthy controls selected from a Chinese population was conducted. *H-RAS *T81C polymorphism was genotyped by Polymerase Chain Reaction-Restriction Fragment Length Polymorphism (PCR-RFLP) assay.

**Results:**

In the healthy controls, the TT, TC and CC genotypes frequencies of *H-RAS *T81C polymorphism, were 79.24%, 19.87% and 0.89%, respectively, and the C allele frequency was 10.83%. Compared with TT genotype, the TC genotype was significantly associated with an increased risk of gastric cancer (adjusted OR = 3.67, 95%CI = 2.21–6.08), while the CC genotype showed an increased risk as well (adjusted OR = 3.29, 95%CI = 0.54–19.86), but it was not statistically significant. In contrast, the frequency of TC genotype was not significantly increased in colon cancer and rectal cancer patients. Further analysis was performed by combining TC and CC genotypes compared against TT genotype. As a result, a statistically significant risk with adjusted OR of 3.65 (95%CI, 2.22–6.00) was found in gastric cancer, while no significant association of *H-RAS *T81C polymorphism with colon cancer and rectal cancer was observed.

**Conclusion:**

These findings indicate, for the first time, that there is an *H-RAS *T81C polymorphism existing in Chinese population, and this SNP might be a low penetrance gene predisposition factor for gastric cancer.

## Background

Gastrointestinal cancer, including cancers of the oesophagus, stomach, small intestine, colon, rectum and liver etc, is a major medical and economic burden worldwide. Although the incidence and mortality of gastrointestinal cancer has been gradually decreasing for decades, some common types of gastrointestinal cancer are steadily in the top five leading cause of new cancer cases and deaths, such as gastric cancer, colon and rectal cancer[1]. Multiple factors have been proposed to play important roles in human carcinogenesis, however, the exact mechanism of gastrointestinal cancer development still remains unclear.

Mammalian cells contain three functional *RAS *proto-oncogenes, known as *H-RAS*, *K-RAS*, and *N-RAS*, which encode small GTP-binding proteins in terms of p21^ras^s. The RAS proteins are GTPases that bind to GTP and GDP nucleotides[2]. The switch between their inactive (GDP-bound) and active (GTP-bound) forms, together with their ability to bind to target proteins, provides the mechanism for the downstream transmission of the cellular signals. Their natural role is to relay extracellularly derived signals to a number of pathways controlling cellular proliferation and differentiation[3]. *RAS *genes have been elucidated as major participants in the development and progression of a series of human tumours, such as gastrointestinal cancer, lung cancer and breast cancer. It was reported that just one point mutation occurring in codon12, 13 or 61 could result in continuous stimulation of cell proliferation or, alternatively, a 5- to 50-fold amplification of the wild type gene[4]. As a result, the codon12, 13 and 61 are also called mutation hotpots. Numerous epidemiological studies on pancreatic, gastric, colorectal and non-small cell lung cancer evaluate the potential role of these mutation hotspots, but the results are still conflicting up to now [5-8].

Besides the mutation hotspots of *H-RAS *mentioned above, another single nucleotide polymorphism at *H-RAS *cDNA position 81 T→C (rs12628), primarily found by Taparowsky et al in 1982[9], was shown to be associated with the risk of human cancers as well. Johne's research indicated that the individuals harbouring the homozygous C-genotype of the *H-RAS *T81C were at an increased risk of bladder cancer [10]. More recently, it was demonstrated that the variant C allele of this genetic polymorphism could increase the risk of oral carcinoma, particularly in male population[11]. However, the number of studies conducted to examine the *H-RAS *T81C polymorphism is not sufficient; moreover, the results of them are controversial yet. Especially, there is a lack of investigation on gastrointestinal cancer, such as gastric, colon and rectal cancer.

Therefore, in the present research, we hypothesize that the *H-RAS *T81C polymorphism may have an effect on the *H-RAS *expression and activity, and ultimately may play a role in modulating the susceptibility to gastrointestinal cancer. In order to verify our hypothesis, a population based case-control study was conducted to investigate the association between the *H-RAS *T81C genotypes and the risk of gastrointestinal cancer in Chinese population. In addition, a meta-analysis was performed to estimate the risk of *H-RAS *T81C polymorphism for cancers.

## Methods

### Study subjects

This population-base case-control study included 296 gastrointestinal cancer patients and 448 healthy controls, and the details of the study population had been described previously[12,13]. In brief, the registry information of this population was initially collected for a cohort study on colorectal cancer in 1989 in Jiashan County, Zhejiang Province, China. Meanwhile a cancer surveillance and registry system covering the whole county was established for reporting new cancer patients of colorectal cancer and all other kinds of cancers. There were no age, gender, or stage restrictions, but patients with other malignant disease in their medical history were excluded. 296 eligible patients with histologically confirmed gastric cancer, colon cancer or rectal cancer reported by the cancer registry system were included as cases in the study. Gastric cancer patients consisted of 64 males and 26 females from 45 to 78 years old, while colon and rectal cancer patients consisted of 105 males and 101 females from 35 to 81 years old. Simultaneously, 448 population controls who did not have a history of cancer were selected randomly and recruited from all permanent residents listed in the cancer registry system during the same period. All the participants were ethnic Han Chinese residents in Jiashan County.

At the beginning of investigation, written informed consent was obtained from each participant, and then they were face-to-face interviewed by professionally trained interviewers using a structured questionnaire, including demographic characteristics, personal habits (cigarette smoking, alcohol drinking, etc) and health factors (family history of cancer at any site including all first- and second-degree relatives of both genders, medical and dietary history, etc). Individuals who smoked ≥1 cigarette per day for over 1 year were defined as smokers, and those consumed ≥1 alcohol drinks per day for over 3 months were considered as drinkers. In addition, a 2 ml venous blood sample was drawn from each subject with the permission and saved in vacuum tube containing sodium citrate anticoagulation. This study was performed with the approval of the Medical Ethical Committee of Zhejiang University School of Medicine. The blood samples were stored at -60°C ultra low temperature freezers for DNA isolation.

### *H-RAS *T81C genotyping

The genomic DNA was extracted from peripheral blood samples using modified salting-out procedure as previously reported[14]. The DNA concentration and purity was measured using BioPhotometer (Eppendorf, Hamburg, Germany) at 260 nm.

For determination of the *H-RAS *genetic polymorphism 81 T→C, Polymerase Chain Reaction – Restriction Fragment Length Polymorphism (PCR-RFLP) assay was performed at the Molecular Epidemiology Laboratory in the Zhejiang University School of Medicine. The sequence of the oligonucleotide primers and the conditions for PCR amplification were reported elsewhere[10]. A 200 bp DNA segment was amplified using forward primer 5'-CTTGGCAGGTGGGGCAGGAGA-3' and reverse primer 5'-GGCACCTGGACGGCGGCGCTAG-3'. The PCR mixture (20 μl) contained 2 μl of 10 × PCR buffer, 2 μl of deoxyribonucleotide triphosphates (dNTPs, 2 mM each), 1.6 μl MgCl_2 _(25 mM each), 0.2 μl of each primer (10 μM each), 0.5 μl genomic DNA (~50 ng) and 0.5 Unit of *Taq *DNA polymerase (Sangon, Shanghai, China). After 5 min of initial denaturation at 94°C in PTC-200 thermal cycler (BioRad, USA), the prepared PCR mixtures were subjected to 35 cycles of denaturation for 30 s at 94°C, annealing for 30 s at 50°C and extension for 30 s at 72°C. A final extension period of 5 min at 72°C was performed to completed the reaction. Subsequently, 6 μl of amplified product was added with 1 μl of 10× digestion buffer containing 3 units DraIII (New England Biolabs, Schwalbach, Germany). After 6 hours digestion at 37°C, the products were separated by electrophoresis on a 2% agarose gel stained with ethidium bromide and observed on an ultraviolet fluorescence imaging system. The CC homozygote with DraIII restriction site was cut into fragments of 145 bp and 55 bp, while the 81TT homozygote presented a single fragment of 200 bp. About 10% of the samples were selected randomly to be identified repeatedly for accuracy, and the results were 100% concordant. The genotype assay described above was done with case/control status blinded to the laboratory technician.

### Statistical analysis

Pearson's χ^2 ^test was used for comparing the distributions of the demographic characteristics, personal habits such as cigarette smoking and alcohol drinking etc, and the distribution of *H-RAS *T81C allele types and genotypes between cases and controls. Hardy-Weinberg equilibrium was tested by a goodness-of-fit χ^2 ^test to compare the observed genotype frequencies within the case-control groups to the anticipated genotype frequencies calculated from the observed allele frequencies. Unconditional logistic regression analysis was performed to calculate the odds ratios (ORs) with 95% confidence intervals (95% CIs) for estimating the association between certain genotype and cancers with adjustment for potential confounding factors, including age (as a continuous variable), gender, smoking, drinking and family history of cancer (as dichotomous variables). *H-RAS *T81C polymorphism was analyzed as dichotomized variable using TT genotype as the reference category. Stratified analyses were used to explore potential gene-environment interactions. A *p *value of less than 0.05 indicated statistical significance. In the meta-analysis, raw data for genotype frequencies, without adjustment, were used for calculation of the estimates of OR. The extent of heterogeneity was examined by the Cochran's χ^2 ^test. The statistical analyses were performed with Statistical Analysis System software version 8.0 (SAS Institute, Cary, NC) and the meta-analysis was carried out using RevMan software version 4.2.

## Results

The demographic characteristics and personal habits of the study subjects were summarized in Table 1. There was no significant difference in gender, age, family history of cancer, smoking and drinking status among colon cancers, rectal cancers and healthy controls. Otherwise, the age, gender and smoking status were significantly different between gastric cancers and controls.

**Table 1:** Distribution of selected characteristics in the study subjects

	**Cases** **No.(%)**				**Controls** **No.(%)**
		
	**Gastrointestinal cancer**	**Gastric cancer**	**Colon cancer**	**Rectal cancer**	
No. of subjects	296	90	93	103	448
Gender					
Male	169(57.09)	64(71.11)	48(51.61)	57(50.44)	223(49.78)
Female	127(42.91)	26(28. 89)	45(48.39)	56(49.56)	225(50.22)
Age					
Mean ± SD	61.76 ± 9.92	64.68 ± 9.01	61.59 ± 8.87	59.57 ± 10.89	60.67 ± 10.92
Smoking					
No	156(52.70)	33(36.67)	53(56.99)	70(61.95)	269(60.04)
Yes	140(47.30)	57(63.33)	40(43.01)	43(38.05)	179(39.96)
Drinking					
No	202(68.24)	56(62.22)	65(69.89)	81(71.68)	322(71.88)
Yes	94(31.76)	34(37.78)	28(30.11)	32(28.32)	126(28.13)
Family history					
No	212(71.62)	68(75.56)	68(73.12)	76(67.26)	338(75.45)
Yes	84(28.38)	22(24.44)	25(26.88)	37(32.74)	110(24.55)
*H-RAS *T81C					
TT	204(68.92)	48(53.33)	71(76.34)	85(75.22)	355(79.24)
TC	88(29.73)	40(44.44)	20(21.51)	28(24.78)	89(19.87)
CC	4(1.35)	2(2.22)	2(2.15)	0(0.00)	4(0.89)
Allele frequencies					
T	496(83.78)	136(75.56)	162(87.10)	198(87.61)	799(89.17)
C	96(16.22)	44(24.44)	24(12.90)	28(12.39)	97(10.83)

The observed genotype distributions of *H-RAS *T81C among cases and controls were shown in Table 1 as well. In healthy controls, the frequencies of TT, TC and CC genotypes were 79.24%, 19.87% and 0.89%, which did not deviate from the Hardy-Weinberg equilibrium (χ^2 ^= 0.375, p = 0.541), while those frequencies were 53.33%, 44.44% and 2.22% in gastric cancer patients, respectively. The frequency of C allele observed in gastric cancers was about 24.44% which was significantly higher than that in controls 10.83% (χ^2 ^= 24.413, p < 0.0001). Meanwhile, the frequencies of C allele in colon cancer and rectal cancer, 12.90% and 12.39% respectively, were also higher than that in controls. However, no significant difference in genotype or allele distribution was found among colon cancer, rectal cancer and controls (data not shown).

Furthermore, crude ORs and adjusted ORs were calculated to evaluate the risk of gastrointestinal cancer. Compared with TT genotype, the TC genotype was significantly associated with a increased risk of gastric cancer (crude OR = 3.32, 95%CI = 2.06–5.37, p < 0.0001), and the CC genotype with more risk, crude OR was 3.69, but it was not statistically significant, this may be due to limited number of CC genotype carriers. The same results were observed after adjustment (shown in Table 2). In contrast, the TC genotype was not significantly increased in colon cancer and rectal cancer patients. Since *H-RAS *is one of proto-oncogene, mutation of which could confer a dominant negative phenotype, and the frequency of the CC genotype is very low (<0.05), further analysis was performed by combining TC and CC genotypes compared against TT genotype. As a result, a statistically significant risk with adjusted OR of 3.65 (95%CI, 2.22–6.00, p < 0.0001) was found in gastric cancer, while no significant association of *H-RAS *T81C polymorphism with colon cancer and rectal cancer was observed. In addition, the effect of *H-RAS *T81C polymorphism was further examined by stratification of age, gender, family history, smoking and drinking status. However, no significant gene-environment interaction in relation to gastric cancer, colon cancer and rectal cancer was found (data not shown).

**Table 2:** H-RAS T81C polymorphism and gastrointestinal cancer risk

**Genotype**	**Gastrointestinal cancer**		**Gastric cancer**		**Colon cancer**		**Rectal cancer**	
	
	**Crude OR** **(95%CI)**	Adjusted OR* (95%CI)	**Crude OR** **(95%CI)**	**Adjusted OR** **(95%CI)**	**Crude OR** **(95%CI)**	**Adjusted OR** **(95%CI)**	**Crude OR** **(95%CI)**	**Adjusted OR** **(95%CI)**
TT	1.00	1.00	1.00	1.00	1.00	1.00	1.00	1.00
TC	1.72(1.22, 2.42)	1.74(1.23, 2.46)	3.32(2.06, 5.37)	3.67(2.21, 6.08)	1.12(0.65, 1.94)	1.15(0.66, 1.99)	1.26(0.76, 2.04)	1.25(0.77, 2.04)
CC	1.74(0.43, 7.03)	1.66(0.41, 6.77)	3.69(0.66, 20.73)	3.29(0.54, 19.86)	2.50(0.45, 13.91)	2.57(0.46, 14.46)	-	-
TC+CC	1.72(1.23, 2.41)	1.74(1.24, 2.44)	3.34(2.08, 5.36)	3.65(2.22, 6.00)	1.18(0.70, 2.01)	1.20(0.71, 2.06)	1.26(0.76, 2.04)	1.25(0.77, 2.04)

*OR adjusted for age, gender, family history, status of cigarette smoking and alcohol drinking

**Figure 1:**
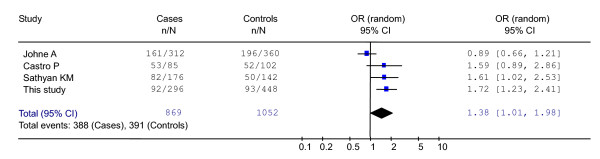
Meta-analysis of H-RAS T81C polymorphism for all cancers.

Three other studies on *H-RAS *T81C polymorphism and cancer risk were available in the literature including bladder, thyroid and oral cancer patients. Because of the small sample size of these studies, we carried out a meta-analysis to obtain an overall assessment of the effects of the *H-RAS *T81C polymorphism on human cancers, which was examined according to *H-RAS *genotype in all published studies and in our patients. As presented in Fig. 1, the test for heterogeneity was not significant (χ^2 ^= 9.87, p = 0.02), suggesting that the random effect model could be used to assess the odds ratio. The pooled odds ratio was 1.38 (95% CI, 1.01–1.98), which was significant.

## Discussion

A multi-factorial model of human carcinogenesis is currently accepted, according to which different dietary and non-dietary factors, including genetic susceptibility, are involved at different stages in the cancer process[15]. *RAS *gene, serving as molecular switches in pivotal processes governing cellular growth and differentiation, have been found to be amplified and over expressed in gastric carcinomas[16,17]. Several molecular alternations in *RAS *gene have been identified such as minisatellites and mutations, however, research on the single nucleotide polymorphism in *RAS *gene was rare [18-20]. In the present study, analyses of the polymorphism of *H-RAS *T81C in gastrointestinal cancer patients were carried out compared with healthy controls. The frequency of C allele in the Chinese healthy population was 10.83%, which was lower than that in Johne's study (26.42% in Germany)[10] and Sathyan's study (20.00% in India)[11]. This observation indicates that the distribution of *H-RAS *T81C polymorphism seems to be genetically different in various ethnics. In the aforementioned study, they reported that *H-RAS *81CC homozygous genotype showed 2-fold risk of bladder cancer and oral cancer. In this study, a statistically significant increased risk for gastric cancer was observed in the TC and CC genotypes combination compared to the TT genotype, and the adjusted OR achieved 3.65, which indicated that *H-RAS *T81C polymorphism is a strongly susceptibility factor for the development of gastric cancer. On the other hand, we did not find any association of *H-RAS *T81C polymorphism with colon cancer and rectal cancer, which derived from the same population.

However, this epidemiological study could not provide the mechanism by which *H-RAS *T81C polymorphism modifies risk of different kinds of cancer. Although *RAS *gene is ubiquitously expressed, the mRNA analysis reveals different tissue expression levels suggesting that *RAS *family members probably are expressed in a tissue specificity fashion[3]. *K-RAS *is mostly expressed in the pancreas, colorectal and non-small cell lung cancer, while *H-RAS *is common in squamous cell carcinomas, bladder carcinomas and renal cancers. *K-RAS *mutations have been found in 15%–68% of sporadic colorectal cancer[21]. Keller et al. reported that endogenous K-RAS resulted in the most dramatic effect of the *RAS *isoforms (K > H or N) in human colorectal cancer cells[22]. However, *K-RAS *mutations are rarely observed in gastric cancer[17,23,24] and increased expression of the *H-RAS *oncogene product was found in gastric cancer[25,26]. Consistent with the tissue specificity hypothesis, our finding suggested that the *H-RAS *gene played a more important role in gastric cancer than in colorectal cancer. Although this polymorphism does not lead to the alternation of RAS protein structure, it affects the cancer susceptibility possibly through linkage disequilibrium with other potential functional variant of *H-RAS*. One of the linkage candidate is a region of variable tandem repeats about 1 kb downstream exon4, with a possible transcriptional enhancer activity[27]. Another associated polymorphic site is hexanucleotide repeat located about 80 bp upstream of the 5'-end of exon1[28]. Recently, it is reported that *H-RAS *T81C might be serve as a marker of other polymorphisms in intron D2 of *H-RAS *that would act as regulators of IDX inclusion[29]. As it was not examined in the present study, it would be interesting to conduct more studies on the linkage of *H-RAS *T81C polymorphism with the other candidate polymorphic sites in order to reveal the underlying mechanism.

It has been suggested that sample size is always an issue of concern in the case-control analysis and statistical power of the analysis would generally increase with increasing size of sample[30]. We only recruited 296 survival cases and the sample size might not be large enough to detect the low penetrance effect of the genes, especially for the SNPs with low frequency. Therefore, we pooled all published data together with ours, and then performed a meta-analysis to examine the association between the *H-RAS *T81C polymorphism and the cancer risk. Overall, the C allele carriers, including TC genotype and CC genotype carriers, have a 38% increased risk of cancer. It is reasonable to believe that the variant of *H-RAS *gene could be associated with cancer risk, and it will be of interest in test if this genetic polymorphism is associated with increased risk of other malignant tumors.

Another limitation should be addressed in this study. *Helicobacter pylori *infection has been defined as a crucial risk factor for gastric cancer by numerous intensive researches, which lead to an improved understanding of the etiology and pathogenesis [31]. Unfortunately, information of *Helicobacter pylori *infection in our study is not available, as a result of that we can not analyze the interaction between *H-RAS *polymorphism and *Hp *infection status.

## Conclusion

In summary, this study provides the evidences that the polymorphism of *H-RAS *T81C may be a risk factor for the development of gastric cancer in a Chinese population. Because this is the first study to report the significant association between *H-RAS *T81C polymorphism and gastric cancer susceptibility, additional studies with large sample size and detailed *Hp *infection information on gastric cancer are warranted in different ethnic populations to confirm our findings.

## Competing interests

The authors declare that they have no competing interests.

## Authors' contributions

YZ participated in the design of the study, SNP genotyping, and drafted the manuscript. MJ, BL participated in SNP genotyping and performed the statistical analysis. XM, KY and QL participated in data collection. KC conceived of the study, and participated in its design and coordination and helped to draft the manuscript. All authors read and approved the final manuscript.

## Pre-publication history

The pre-publication history for this paper can be accessed here:



## References

[B1] Parkin DM, Bray F, Ferlay J, Pisani P (2005). Global cancer statistics, 2002. CA: a cancer journal for clinicians.

[B2] Malumbres M, Pellicer A (1998). RAS pathways to cell cycle control and cell transformation. Front Biosci.

[B3] Spandidos DA, Sourvinos G, Tsatsanis C, Zafiropoulos A (2002). Normal ras genes: their onco-suppressor and pro-apoptotic functions (review). Int J Oncol.

[B4] Macaluso M, Russo G, Cinti C, Bazan V, Gebbia N, Russo A (2002). Ras family genes: an interesting link between cell cycle and cancer. J Cell Physiol.

[B5] Victor T, Du Toit R, Jordaan AM, Bester AJ, van Helden PD (1990). No evidence for point mutations in codons 12, 13, and 61 of the ras gene in a high-incidence area for esophageal and gastric cancers. Cancer Res.

[B6] Deng GR, Liu XH, Wang JR (1991). Correlation of mutations of oncogene C-Ha-ras at codon 12 with metastasis and survival of gastric cancer patients. Oncogene Res.

[B7] Hao Y, Zhang J, Lu Y, Yi C, Qian W, Cui J (1998). The role of ras gene mutation in gastric cancer and precancerous lesions. J Tongji Med Univ.

[B8] Feng J, Hua F, Shuo R, Chongfeng G, Huimian X, Nakajima T, Subao W, Tsuchida N (2001). Upregulation of non-mutated H-ras and its upstream and downstream signaling proteins in colorectal cancer. Oncol Rep.

[B9] Taparowsky E, Suard Y, Fasano O, Shimizu K, Goldfarb M, Wigler M (1982). Activation of the T24 bladder carcinoma transforming gene is linked to a single amino acid change. Nature.

[B10] Johne A, Roots I, Brockmoller J (2003). A single nucleotide polymorphism in the human H-ras proto-oncogene determines the risk of urinary bladder cancer. Cancer Epidemiol Biomarkers Prev.

[B11] Sathyan KM, Nalinakumari KR, Abraham T, Kannan S (2006). Influence of single nucleotide polymorphisms in H-Ras and cyclin D1 genes on oral cancer susceptibility. Oral Oncol.

[B12] Chen K, Jin M, Zhu Y, Jiang Q, Yu W, Ma X, Yao K (2006). Genetic polymorphisms of the uridine diphosphate glucuronosyltransferase 1A7 and colorectal cancer risk in relation to cigarette smoking and alcohol drinking in a Chinese population. J Gastroenterol Hepatol.

[B13] Fan C, Jin M, Chen K, Zhang Y, Zhang S, Liu B (2007). Case-only study of interactions between metabolic enzymes and smoking in colorectal cancer. BMC cancer.

[B14] Nasiri H, Forouzandeh M, Rasaee MJ, Rahbarizadeh F (2005). Modified salting-out method: high-yield, high-quality genomic DNA extraction from whole blood using laundry detergent. J Clin Lab Anal.

[B15] Kelley JR, Duggan JM (2003). Gastric cancer epidemiology and risk factors. Journal of clinical epidemiology.

[B16] Crespo P, Leon J (2000). Ras proteins in the control of the cell cycle and cell differentiation. Cell Mol Life Sci.

[B17] Yoo J, Park SY, Robinson RA, Kang SJ, Ahn WS, Kang CS (2002). ras Gene mutations and expression of Ras signal transduction mediators in gastric adenocarcinomas. Arch Pathol Lab Med.

[B18] Gonzalez CA, Sala N, Capella G (2002). Genetic susceptibility and gastric cancer risk. Int J Cancer.

[B19] Hiyama T, Haruma K, Kitadai Y, Masuda H, Miyamoto M, Tanaka S, Yoshihara M, Shimamoto F, Chayama K (2002). K-ras mutation in helicobacter pylori-associated chronic gastritis in patients with and without gastric cancer. Int J Cancer.

[B20] Langdon JA, Armour JA (2003). Evolution and population genetics of the H-ras minisatellite and cancer predisposition. Hum Mol Genet.

[B21] Takayama T, Miyanishi K, Hayashi T, Sato Y, Niitsu Y (2006). Colorectal cancer: genetics of development and metastasis. Journal of gastroenterology.

[B22] Keller JW, Franklin JL, Graves-Deal R, Friedman DB, Whitwell CW, Coffey RJ (2007). Oncogenic KRAS provides a uniquely powerful and variable oncogenic contribution among RAS family members in the colonic epithelium. J Cell Physiol.

[B23] van Rees BP, Musler A, Caspers E, Drillenburg P, Craanen ME, Polkowski W, Chibowski D, Offerhaus GJ (1999). K-ras mutations in gastric stump carcinomas and in carcinomas from the non-operated stomach. Hepato-gastroenterology.

[B24] Arber N, Shapira I, Ratan J, Stern B, Hibshoosh H, Moshkowitz M, Gammon M, Fabian I, Halpern Z (2000). Activation of c-K-ras mutations in human gastrointestinal tumors. Gastroenterology.

[B25] Ohuchi N, Hand PH, Merlo G, Fujita J, Mariani-Costantini R, Thor A, Nose M, Callahan R, Schlom J (1987). Enhanced expression of c-Ha-ras p21 in human stomach adenocarcinomas defined by immunoassays using monoclonal antibodies and in situ hybridization. Cancer Res.

[B26] Fujita K, Ohuchi N, Yao T, Okumura M, Fukushima Y, Kanakura Y, Kitamura Y, Fujita J (1987). Frequent overexpression, but not activation by point mutation, of ras genes in primary human gastric cancers. Gastroenterology.

[B27] Trepicchio WL, Krontiris TG (1992). Members of the rel/NF-kappa B family of transcriptional regulatory proteins bind the HRAS1 minisatellite DNA sequence. Nucleic Acids Res.

[B28] Kotsinas A, Gorgoulis VG, Zacharatos P, Mariatos G, Kokotas S, Liloglou T, Ikonomopoulos J, Zoumpourlis V, Kyroudi A, Field JK, Asimacopoulos PJ, Kittas C (2001). Additional characterization of a hexanucleotide polymorphic site in the first intron of human H-ras gene: comparative study of its alterations in non-small cell lung carcinomas and sporadic invasive breast carcinomas. Cancer genetics and cytogenetics.

[B29] Castro P, Soares P, Gusmao L, Seruca R, Sobrinho-Simoes M (2006). H-RAS 81 polymorphism is significantly associated with aneuploidy in follicular tumors of the thyroid. Oncogene.

[B30] Zhang HT (2005). Int7G24A variant of the TGFBR1 gene and cancer risk: a meta-analysis of three case-control studies. Lung cancer (Amsterdam, Netherlands).

[B31] Uemura N, Okamoto S, Yamamoto S, Matsumura N, Yamaguchi S, Yamakido M, Taniyama K, Sasaki N, Schlemper RJ (2001). Helicobacter pylori infection and the development of gastric cancer. N Engl J Med.

